# Somatic Disease in Survivors of Childhood Malignant Bone Tumors in the Nordic Countries

**DOI:** 10.3390/cancers13184505

**Published:** 2021-09-07

**Authors:** Camilla Pedersen, Catherine Rechnitzer, Elisabeth Anne Wreford Andersen, Line Kenborg, Filippa Nyboe Norsker, Andrea Bautz, Thomas Baad-Hansen, Laufey Tryggvadottir, Laura-Maria Madanat-Harjuoja, Anna Sällfors Holmqvist, Lars Hjorth, Henrik Hasle, Jeanette Falck Winther, on behalf of the ALiCCS Study Group

**Affiliations:** 1 Danish Cancer Society Research Center, 2100 Copenhagen, Denmark; elian@cancer.dk (E.A.W.A.); kenborg@cancer.dk (L.K.); fnorsker@hotmail.com (F.N.N.); andrea.bautz@gmail.com (A.B.); jeanette@cancer.dk (J.F.W.); 2 Department of Pediatrics and Adolescent Medicine, Copenhagen University Hospital, 2100 Copenhagen, Denmark; catherine.rechnitzer@regionh.dk; 3 Department of Orthopedic Surgery, Sarcoma Centre of Aarhus University Hospital, 8200 Aarhus, Denmark; thombaad@rm.dk; 4 The Icelandic Cancer Registry, 105 Reykjavik, Iceland; laufeyt@krabb.is; 5 Faculty of Medicine, University of Iceland, 102 Reykjavik, Iceland; 6 The Finnish Cancer Registry, 00130 Helsinki, Finland; Laura.Madanat@cancer.fi; 7 Department of Clinical Sciences Lund, Lund University, 222 41 Lund, Sweden; anna.sallfors-holmqvist@med.lu.se (A.S.H.); Lars.Hjorth@skane.se (L.H.); 8 Department of Pediatric Hematology and Oncology, Skane University Hospital, 221 85 Lund, Sweden; 9 Department of Pediatrics, Aarhus University Hospital, 8200 Aarhus, Denmark; hasle@dadlnet.dk; 10 Department of Clinical Medicine, Faculty of Health, Aarhus University and University Hospital, 8200 Aarhus, Denmark

**Keywords:** childhood malignant bone tumors, survivorship, late effects, somatic disease, cohort study

## Abstract

**Simple Summary:**

The treatment of osteosarcoma and Ewing sarcoma, the two major types of malignant bone tumors in children, has progressed considerably during the last decades, with more patients becoming long-term survivors. This improvement has resulted in an increasing number of patients with long-term adverse health consequences from the life-saving treatment. The aim of this study was to provide a detailed, comprehensive overview of somatic diseases that require hospitalization in long-term survivors of osteosarcoma and Ewing sarcoma. This study contributes new insights into the risk of somatic late effects in survivors of osteosarcoma and Ewing sarcoma which are urgently requested by pediatric oncologists, researchers, and by survivors and their families. The study provides an essential basis for the development of preventive intervention strategies and for optimal patient counseling and follow-up care, which all contribute to improving the health and quality of life in survivors.

**Abstract:**

Survivors of malignant bone tumors in childhood are at risk of long-term adverse health effects. We comprehensively reviewed cases of somatic diseases that required a hospital contact in survivors of osteosarcoma and Ewing sarcoma. In a population-based cohort study, 620 five-year survivors of osteosarcoma (n = 440) or Ewing sarcoma (n = 180), diagnosed before the age of 20 years in Denmark, Finland, Iceland, and Sweden during 1943–2008, were followed in the national hospital registers. Overall rates of hospital contacts for any somatic disease and for 12 main diagnostic groups and 120 specific disease categories were compared with those in a matched comparison cohort (n = 3049) randomly selected from the national population registers. The rate of hospital contact for any somatic disease was 80% higher in survivors of malignant bone tumors than in comparisons and remained elevated up to 30 years after diagnosis. The rate of hospital contacts was higher after Ewing sarcoma (rate ratio (RR) 2.24; 95% confidence interval (CI) 1.76–2.85) than after osteosarcoma (RR 1.67; 95% CI 1.41–1.98). Elevated rates were observed for 11 main diagnostic groups, including infections, second malignant neoplasms, and diseases of the skin, bones, and circulatory, digestive, endocrine, and urinary systems. Survivors of malignant bone tumors in childhood are at increased risk of somatic diseases many years after diagnosis. This comprehensive study contributes new insight into the risk of late effects in survivors of osteosarcoma and Ewing sarcoma, which is an essential basis for optimal patient counseling and follow-up care.

## 1. Introduction

Osteosarcoma and Ewing sarcoma are the commonest primary malignant bone tumors in children and adolescents, accounting for approximately 6% of all childhood cancers [1,2]. The commonest site for both tumors is the extremities, but more Ewing sarcomas than osteosarcomas occur in the axial skeleton [1,2]. Chemotherapy and surgery are the primary therapy for osteosarcoma, as the tumor is not responsive to radiation at conventional doses [2]. The chemotherapeutic agents used in the treatment of osteosarcoma include cisplatin, doxorubicin, and high-dose methotrexate, and those for Ewing sarcoma are mainly vincristine, ifosfamide, doxorubicin, etoposide, actinomycin D, and cyclophosphamide [1,2,3]. Ewing sarcoma is controlled locally with surgery, radiation therapy, or a combination of the two [1]. Previously, most bone tumors in the extremities were managed by amputation, however, advances in high-dose chemotherapy and in surgical techniques have made limb salvage the leading surgical procedure [2].

Survival after osteosarcoma and Ewing sarcoma diagnosed in childhood improved significantly with the introduction of chemotherapy in the 1970s, from 25% and 10% before use of chemotherapy, respectively, to 60–70% [1,2]. Improved survival has resulted in increasing numbers of patients with long-term adverse health effects of the tumors and their treatments.

The majority of previous studies of somatic late effects in survivors of malignant bone tumors in childhood addressed either exclusively the overall risk of adverse health effects and health care use [4,5,6] or the risk of one or a few specific somatic late effects, especially secondary cancers [7,8,9,10,11,12,13,14,15,16,17,18,19,20]. Only a few studies [21,22,23] evaluated the risks of survivors of childhood malignant bone tumors for hospitalization for conditions in a wide range of main diagnostic groups, including infections, neoplasms, and diseases of the endocrine, circulatory, nervous, respiratory, digestive, and genitourinary systems. None of the studies reported risk estimates for subtypes of malignant bone tumors or for specific underlying disease categories.

In order to provide a detailed, comprehensive overview of somatic diseases that require hospital contact in long-term survivors of malignant bone tumors, we followed all children with a malignant bone tumor diagnosed in the Nordic countries. High-quality nationwide hospital registers and population registers allow long-term follow-up for medically verified diagnoses, with virtually no loss to follow-up. This is the first population-based cohort study that provides relative rates of hospital contacts for the full range of main diagnostic groups in survivors of osteosarcoma and Ewing sarcoma and relative and absolute rates for 120 specific disease categories in survivors of childhood malignant bone tumors.

## 2. Materials and Methods

### 2.1. Survivors of Malignant Bone Tumors and Comparison Cohort

The study was conducted within the large Nordic population-based research program Adult Life after Childhood Cancer in Scandinavia (ALiCCS) [24], which comprises all children in the Nordic countries with cancer diagnosed before the age of 20 years from the start of the national cancer registries in the 1940s and 1950s until 2008. In accordance with the International Classifications of Childhood Cancer (ICCC) [25,26], 1661 children were registered with a primary malignant bone tumor in Denmark, Finland, Iceland, or Sweden in the ALiCCS cohort. Subsequently, 101 children were excluded because the morphology code of their tumor was not for a bone tumor or was for an unverified cancer, leaving 1560 children with a malignant bone tumor.

Since the start of population registries in the Nordic countries (Denmark, 1968; Finland, 1971; Iceland, 1955; Sweden, 1968), all residents have been assigned a unique personal identification number which allows accurate linkage of information across nationwide registers of health, migration, and vital status for all residents of the Nordic countries, with virtually no loss to follow-up.

**Figure 1:**
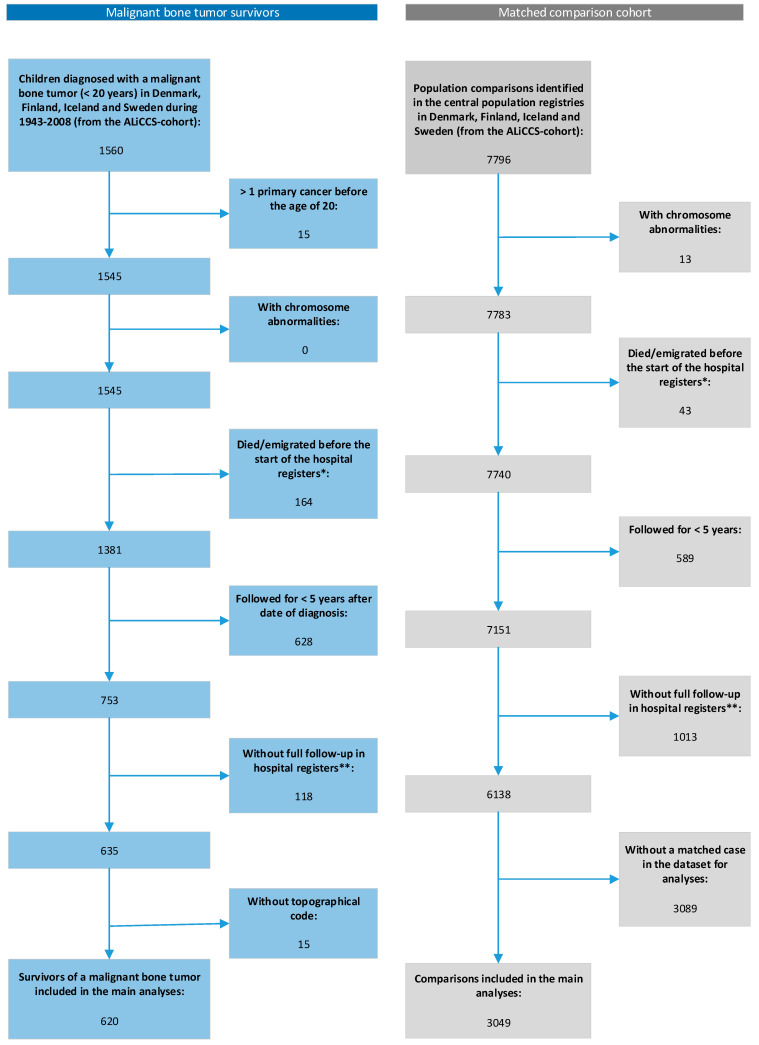
Flow diagram showing exclusions from the cohort of malignant bone tumor survivors and the matched comparisons cohort. * Information on inpatient admissions was available from the start of the hospital registers (Denmark, 1977; Finland, 1983; Iceland, 1999; Sweden, 1968), and outpatient visits were available for Denmark from 1995 and for Sweden from 2001. ** Individuals diagnosed more than 5 years before the start of the hospital registers were excluded to avoid a gap in information on the history of hospital contacts and to ensure accurate rates of hospital contacts.

Five comparisons for each cancer survivor were selected at random from the population registers and matched to survivors by sex, age, and country (Denmark, Finland, and Iceland) or county (Sweden). The vital and migration status of survivors and comparisons was obtained from the national population registers. Comparisons had to be alive on the date of the cancer diagnosis of the corresponding survivor and without a cancer diagnosis before 20 years of age. A total of 7796 comparisons from the general population were matched to the 1560 survivors of a malignant bone tumor. After relevant exclusions, the final dataset comprised 620 five-year survivors and 3049 comparisons (Figure 1).

In order to stratify risk estimates by the major types of bone tumor, we created two sub-cohorts consisting of survivors of osteosarcoma and of Ewing sarcoma (Supplemental Table S1, online). Topographical sites were obtained from the cancer registries and grouped into the categories “extremities” and “axial skeleton”.

### 2.2. Hospital Contacts for Somatic Diseases

By linking the cohort to the national hospital registers, we obtained a full history of inpatient admissions and outpatient visits for somatic diseases (“hospital contacts”). The hospital registers contain the dates of admission and discharge, a primary discharge diagnosis, and supplementary diagnoses coded according to the International Classification of Diseases (ICD) [27,28,29,30]. Registration by treating physicians is mandatory. To estimate the disease burden among survivors, we grouped the diagnoses into 12 main groups according to the ICD, further subdivided into 120 disease categories (Supplemental Table S2, online). We included only the primary discharge diagnosis in the analyses. Information on secondary cancers in survivors and primary cancers in comparisons was obtained from the cancer registries, as the hospital registers do not distinguish between primary and secondary cancers.

### 2.3. Statistical Analyses

Follow-up started 5 years after the date of cancer diagnosis for survivors and the corresponding date for comparisons and ended at the date of death, migration, or the end of the study (Denmark, 31 October 2010; Finland, 31 December 2012; Iceland, 31 December 2008; Sweden, 31 December 2009), whichever came first. As the comparisons were restricted to individuals without a childhood cancer, follow-up for cancer (second malignant neoplasm in survivors and first primary cancer in comparisons) did not start until they were 20 years of age. Only the first diagnosis in each of the 120 disease categories was retained. As the disease categories were grouped into 12 main groups, individuals could have several diagnoses in each main group.

Rate ratios (RRs) and 95% confidence intervals (CIs) for hospital contacts were calculated in marginal rates models [31] with age as the underlying time scale and allowing each individual to have several hospital contacts during follow-up. To account for recurrent hospital contacts for some individuals, a sandwich estimator was used for variance. We compared the overall rates of hospital contacts for any disease in the cohort of all survivors and in survivors of osteosarcoma and Ewing sarcoma with the rate in the matched comparisons. Rates were stratified by sex, age at diagnosis (0–14; 15–19 years), period of diagnosis (1961–1969; 1970–1979; 1980–1989; ≥1990), years since diagnosis (5–9; 10–19; ≥20), and cancer site (extremities, axial skeleton). RRs for hospital contacts were also calculated for the 12 main diagnostic groups. Estimates were calculated for all survivors of a malignant bone tumor and for osteosarcoma and Ewing sarcoma separately. RRs and rate differences for the 120 disease categories were calculated in unadjusted Poisson models for all survivors of a malignant bone tumor and matched comparisons.

The cumulative incidence of first hospital contact was estimated by accounting for death as a competing event and with year since diagnosis or entry as the underlying time scale. To compare the disease burden of survivors and of comparisons, we calculated the mean cumulative count of all first hospital contacts in the 120 disease categories. The cumulative incidence and the mean cumulative count were calculated for all survivors of a malignant bone tumor and for survivors of osteosarcoma and Ewing sarcoma separately.

Statistical analyses were performed in R version 3.6.1 and Stata version 14.2. The study was approved by the national bioethics committees, the data protection authorities, or the national institute for health and welfare in the respective countries.

## 3. Results

The survivor cohort consisted of 440 (71.0%) patients with osteosarcoma and 180 (29.0%) with Ewing sarcoma. Table 1 summarizes key characteristics of survivors and comparisons.

A total of 537 hospital contacts were observed among the 620 survivors of a childhood malignant bone tumor during follow-up, and survivors had an 80% higher rate of hospital contacts for any disease than comparisons (RR 1.80; 95% CI 1.56–2.08; Table 2). After stratification by the two major types of malignant bone tumor, a statistically significantly higher rate (*p* = 0.044) was seen for survivors of Ewing sarcoma (RR 2.24; 95% CI 1.76–2.85) than of osteosarcoma (RR 1.67; 95% CI 1.41–1.98).

The rate of hospital contacts for any disease was higher among men (RR = 2.08) than women (RR = 1.54; Table 2). The rate of hospital contacts remained elevated throughout follow-up, being highest 5–9 years and ≥20 years after the diagnosis.

**Figure 2:**
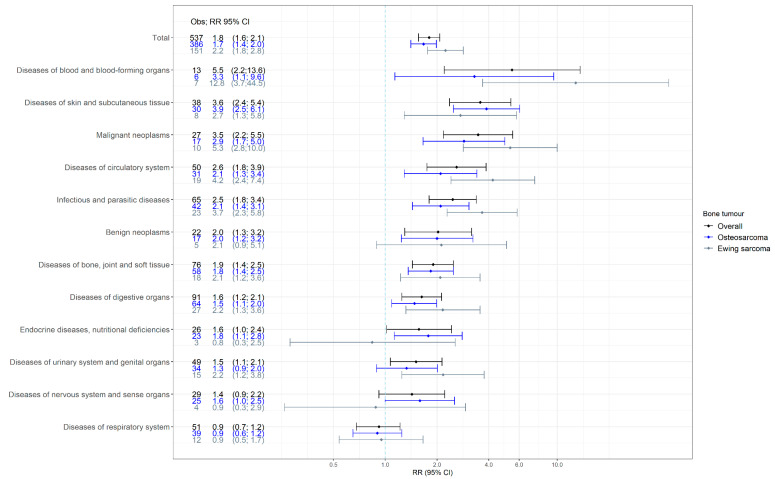
Rate ratios (RRs) and 95% confidence intervals (CIs) for hospital contacts for the 12 main diagnostic groups among survivors of a malignant bone tumor and stratified by osteosarcoma and Ewing sarcoma. Obs: observed number of hospital contacts by survivors.

The relative rates of hospital contacts for survivors of childhood malignant bone tumors were increased for 11 of the 12 main disease groups (statistically significantly in 10 of 12) (Figure 2). The rate of hospital contacts for respiratory diseases (RR 0.92; 95% CI 0.68–1.22) was similar to that in matched comparisons. The highest rates were seen for diseases of the blood and blood-forming organs (RR 5.46; 95% CI 2.20–13.59), followed by diseases of the skin and subcutaneous tissue (RR 3.57; 95% CI 2.37–5.38) and malignant neoplasms (RR 3.47; 95% CI 2.18–5.52). In survivors of osteosarcoma, the highest rate was seen for diseases of the skin and subcutaneous tissue (RR 3.88), followed by diseases of the blood and blood-forming organs (RR 3.30) and malignant neoplasms (RR 2.87). In survivors of Ewing sarcoma, the three highest rates were observed for diseases of the blood and blood-forming organs (RR 12.81), malignant neoplasms (RR 5.33), and diseases of the circulatory system (RR 4.22).

**Figure 3:**
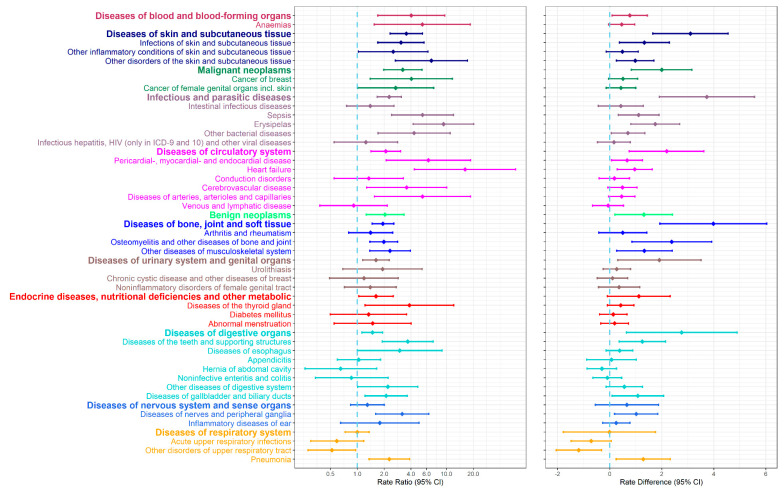
Rate ratios of hospital contacts, rate differences per 1000 person years, and 95% confidence intervals (CIs) for first diagnosis of each of the 120 specific disease categories for survivors compared with the matched comparisons. Only diseases for which survivors had five or more hospital contacts are shown.

The RRs for hospital contacts for the specific disease categories with more than five hospital contacts observed among the survivors are shown in Figure 3. The highest relative rate was observed for heart failure (RR 16), which was observed, however, in only nine survivors (Supplemental Table S3 is a comprehensive list of numbers of hospital contacts, RRs, and 95% CIs). Other disease categories for which the relative rate was >5 were sepsis, erysipelas, anemias, pericardial-, myocardial-, and endocardial disease, diseases of arteries, arterioles, and capillaries, and other disorders of the skin and subcutaneous tissue. Stratification of the overall relative rate for respiratory diseases by specific disease category showed higher rates for pneumonia but lower rates for acute upper respiratory infections and other disorders of the upper respiratory tract.

Figure 3 also provides estimates of the rate difference. None of the estimates were >4 per 1000 person-years. The main diagnostic group with the highest observed rate difference was diseases of bone, joint, and soft tissue, for which survivors of a malignant bone tumor had 3.98 more first hospital contacts per 1000 person-years than the comparisons. The highest rate difference of 2.38 per 1000 person-years was seen for osteomyelitis and other diseases of bone and joint.

**Figure 4:**
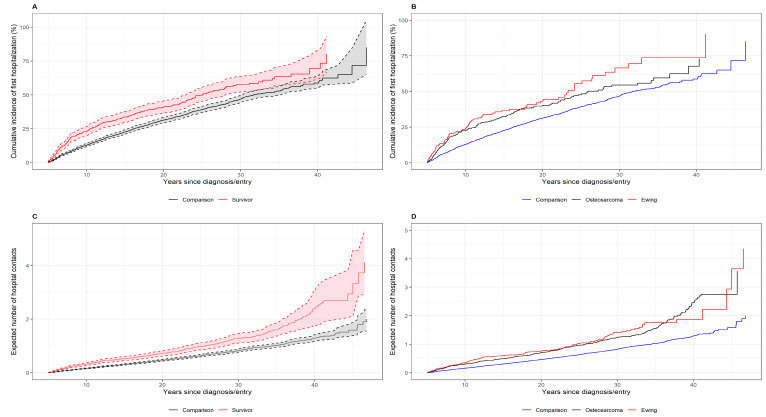
Cumulative incidence of first hospital contact and 95% confidence intervals (CIs) by time since diagnosis or entry for survivors and comparisons (panels **A** and **B**). Expected numbers of hospital contacts (mean cumulative count) with 95% CI for survivors and their matched comparisons by time since diagnosis or entry (panels **C** and **D**).

At 25 years after diagnosis, 49.8% (95% CI 44.6–55.0%) of all survivors of a malignant bone tumor and 39.3% (95% CI 36.9–41.6%) of the comparisons had had at least one hospital contact for any disease (Figure 4A), and the elevated risk persisted up to 30 years after diagnosis. Stratification by type of bone tumor showed that 55.5% of Ewing sarcoma survivors and 47.6% of osteosarcoma survivors had had at least one hospital contact for any disease 25 years after diagnosis (Figure 4B).

Figure 4C,D shows the mean cumulative count, including all first hospital contacts for the 120 disease categories. By 25 years after diagnosis, survivors of a malignant bone tumor had had an average of 0.98 hospital contacts, and comparisons had had 0.64.

## 4. Discussion

This population-based cohort study of 620 survivors of a childhood malignant bone tumor in four countries showed an 80% higher rate of hospital contacts for somatic disease among survivors than matched comparisons. Survivors of Ewing sarcoma had higher rates of hospital contacts than survivors of osteosarcoma. The relative rates of hospital contacts by survivors were higher than those by comparisons for 11 of 12 main diagnostic groups.

Our finding of a 1.8-times increased risk for a hospital contact for any disease in survivors of malignant bone tumors is in line with the results of a study within the North American Childhood Cancer Survivor Study (CCSS) of self-reported hospitalization by survivors of childhood cancer, which gave a standardized incidence ratio (SIR) of 1.6 for bone cancer survivors (n = 854) [22]. In a cohort study of hospitalization of five-year survivors of childhood, adolescent, and young adult cancer in Scotland [21], the standardized hospitalization rate ratio (SHR) for 188 survivors of bone cancer was 3.8 for all diagnoses combined. The increased risks for hospital contacts for infections and diseases of the circulatory, endocrine, nervous, digestive, and genitourinary systems observed in our study are in accordance with the results of both these studies, although they reported markedly higher relative risks for second malignant neoplasms (SHR 10.3 and SIR 6.8) than the RR of 3.5 observed in our study. The lower rate of second malignant neoplasms seen in our study may be influenced by the inclusion of second malignant neoplasms occurring only after the age of 20 years.

To the best of our knowledge, this is the first population-based comparison of the risk of hospital contacts in survivors of osteosarcoma and Ewing sarcoma and to provide estimates of the relative and absolute rate of hospital contacts for 120 disease categories in survivors of malignant bone tumors. The rate of hospital contacts was higher among survivors of Ewing sarcoma than of osteosarcoma for most of the main diagnostic groups, including second malignant neoplasms, diseases of the circulatory system, infections, and diseases of the blood and blood-forming organs. The higher rates seen in survivors of Ewing sarcoma is probably due to the more frequent location in the axial skeleton and the use of radiation therapy for local control. Radiation of the chest is a well-established risk factor for cardiovascular disease [32] and for second malignancies such as sarcomas occurring in the radiation field [13].

Heart failure was the specific disease category with the highest relative rate, which is most likely attributable to the use of the cardiotoxic drug doxorubicin in the treatment of both osteosarcoma and Ewing sarcoma. The elevated risk for pericardial, myocardial, and endocardial disease is also likely to be related to doxorubicin and/or radiation therapy with the heart in the radiation field. Both limb-salvage surgery and amputation often require several surgical revisions over time to maintain a functional limb or in case of prosthesis-related infection, loosening, or breakage [1,2]. The highest rate difference was seen for osteomyelitis and other diseases of bone and joint, which is probably related to complications of endoprosthesis.

Cancer of the breast was the second malignant neoplasm with the highest relative rate in female survivors in our study, which could be related to both radiotherapy to the chest and chemotherapy. The majority of second malignancies reported in survivors of Ewing sarcoma are acute myeloid leukemia, myelodysplastic syndrome and sarcomas in the radiation field and secondary malignancies observed in osteosarcomas include leukemia, breast, lung, kidney, central nervous system, and colon cancer [2]. Most cases of leukemia in survivors of malignant bone tumors occur within five years from diagnosis [2], which explains why they were not captured in our study. The highest rate of hospital contacts of survivors was seen for diseases of the blood and blood-forming organs, especially anemias, which might be in line with the fact that hematological abnormalities in survivors of osteosarcoma have been previously documented even a long time after treatment [33].

The similar rates of hospital contacts of both survivors and comparisons for the main diagnostic group respiratory diseases were a result of a higher rate of pneumonia, but lower rates of acute upper respiratory infections and other disorders of the upper respiratory tract. Both the Scottish study [21] and the CCSS study [22] found elevated estimates of 2.1 and 2.9 for respiratory and pulmonary diseases but provided no information on the relative risks for specific respiratory diseases.

The major strengths of this study include the population-based design and the high-quality nationwide hospital registers in the four countries, with medically verified discharge diagnoses. Moreover, as 70% of the survivors in this study who were alive five years after diagnosis survived to 40 years of age, we were able to investigate long-term somatic late effects. The matched comparison cohort was selected at random from the Nordic population registers, providing unbiased rates of hospital contacts by the general population. In addition, the survivor cohort was restricted to those with full follow-up in the national hospital registers to prevent gaps in information and ensure accurate rates of hospital contacts.

The major limitation of the study is the lack of information on treatment, which prevented investigation of the association between specific cancer treatment and risk of somatic late effects. Further, only morbidity serious enough to require a hospital contact was included, and less serious morbidity treated by a general practitioner in primary healthcare was not captured. Some of the relative risk estimates for Ewing sarcoma had high uncertainty because of the few survivors and events in this sub-group.

## 5. Conclusions

Survivors of malignant bone tumors are at increased risk of hospital contacts for somatic diseases, and the elevated risk persists up to 30 years after diagnosis. The rates of hospital contacts were increased for 11 of 12 main diagnostic groups. Survivors of Ewing sarcoma had higher rates of hospital contacts than survivors of osteosarcoma. This comprehensive population-based study provides detailed information on, and new insight into, the risk of late effects in survivors of childhood malignant bone tumors, which serves as an essential basis for patient counseling and for ensuring optimal follow-up care.

## Figures and Tables

**Table 1:** Characteristics of the cohort of malignant bone tumor survivors overall, of the sub-cohorts of osteosarcoma and Ewing sarcoma, and of the matched comparison cohort.

	5-Year Survivors of Malignant Bone Tumors			Comparisons
	All N (%)	Osteosarcoma ^a^ N (%)	Ewing Sarcoma N (%)	N (%)
Overall	620 (100.0) ^cb^	440 (100.0)	180 (100.0)	3049 (100.0) ^c^
Country				
Denmark and Iceland	145 (23.4)	92 (20.9)	53 (29.4)	699 (22.9)
Finland	176 (28.4)	146 (33.2)	30 (16.7)	870 (28.5)
Sweden	299 (48.2)	202 (45.9)	97 (53.9)	1480 (48.5)
Period of diagnosis				
1961–1979	129 (20.8)	90 (20.5)	39 (21.7)	-
1980–1989	152 (24.5)	112 (25.5)	40 (22.2)	-
1990–2008	339 (54.7)	238 (54.1)	101 (56.1)	-
Sex				
Male	338 (54.5)	234 (53.2)	104 (57.8)	1666 (54.6)
Female	282 (45.5)	206 (46.8)	76 (42.2)	1383 (45.4)
Age at diagnosis				
Mean (SD)	13.7 (4.0)	14.2 (3.7)	12.3 (4.4)	-
0–14	347 (56.0)	226 (51.4)	121 (67.2)	-
15–19	273 (44.0)	214 (48.6)	59 (32.8)	-
Cancer site				
Extremities	485 (78.2)	382 (86.8)	103 (57.2)	-
Axial skeleton	135 (21.8)	58 (13.2)	77 (42.8)	-

SD: standard deviation; ^a^ Including osteosarcoma-like bone sarcomas treated as osteosarcoma; ^b^ The 620 five-year survivors of a malignant bone tumor diagnosed in childhood or adolescence were followed in the national hospital registers for a median of 12.5 years (range: 0–42 years), accruing 8854 person-years. ^c^ The 3049 comparisons were followed-up for a median of 13.8 years (range: 0–42 years) and accrued 47,478 person-years.

**Table 2:** Rate ratios of hospital contacts (RRs) and 95% confidence intervals (CIs) for all survivors of malignant bone tumors and for the sub-cohorts of survivors of osteosarcoma and Ewing sarcoma compared with the matched comparison cohort, stratified by sex, age at diagnosis, year of diagnosis, years since diagnosis, and cancer site.

	All Survivors of Malignant Bone Tumors		*p*-Value	Osteosarcoma		Ewing Sarcoma	
	Observed Number of Hospital Contacts	RR (95% CI)		Observed Number of Hospital Contacts	RR (95% CI)	Observed Number of Hospital Contacts	RR (95% CI)
Overall	537	1.80 (1.56 to 2.08)		386	1.67 (1.41 to 1.98)	151	2.24 (1.76 to 2.85)
Test for same effect in survivors of osteosarcoma and Ewing sarcoma			0.044				
Sex							
Men	302	2.08 (1.69 to 2.56)		210	1.91 (1.50 to 2.42)	92	2.63 (1.85 to 3.73)
Women	235	1.54 (1.26 to 1.87)		176	1.46 (1.16 to 1.84)	59	1.82 (1.37 to 2.42)
Test for interaction *			0.038				
Age at diagnosis (years)							
0–14	299	1.64 (1.36 to 1.98)		198	1.51 (1.20 to 1.89)	101	1.99 (1.48 to 2.67)
15–19	238	2.05 (1.63 to 2.58)		188	1.90 (1.47 to 2.45)	50	2.93 (1.89 to 4.54)
Test for interaction *			0.138				
Period of diagnosis							
1961–1969	54	3.54 (1.84 to 6.80)		44	3.13 (1.48 to 6.59)	10	8.19 (5.37 to 12.47)
1970–1979	174	1.47 (1.15 to 1.86)		117	1.30 (0.98 to 1.71)	57	1.99 (1.34 to 2.95)
1980–1989	163	1.69 (1.31 to 2.17)		127	1.65 (1.23 to 2.21)	36	1.87 (1.30 to 2.67)
≥1990	146	2.17 (1.69 to 2.79)		98	2.02 (1.53 to 2.67)	48	2.58 (1.63 to 4.08)
Test for interaction *			0.025				
Time since diagnosis (years)							
5–9	185	2.14 (1.73 to 2.64)		127	1.94 (1.53 to 2.46)	58	2.76 (1.90 to 4.03)
10–19	160	1.44 (1.16 to 1.79)		113	1.34 (1.03 to 1.74)	47	1.74 (1.26 to 2.41)
≥20	192	1.92 (1.50 to 2.45)		146	1.80 (1.36 to 2.39)	46	2.40 (1.59 to 3.62)
Test for interaction *			0.016				
Cancer site							
Extremities	412	1.75 (1.48 to 2.08)		320	1.66(1.37 to 2.01)	92	2.16 (1.57 to 2.98)
Axial skeleton	125	1.98 (1.56 to 2.53)		66	1.73(1.27 to 2.37)	59	2.37 (1.67 to 3.36)
Test for interaction *			0.394				

* Test for common effect, whether the effect of being a survivor of a malignant bone tumor is the same in men and women, across different age groups at diagnosis, different period of diagnosis, different time intervals since diagnosis, or in different cancer sites.

## Data Availability

Data are not available due to Danish legislation. However, the study group welcomes collaboration with other researchers using our registry data. For further information regarding collaboration, please contact Jeanette Falck Winther, (jeanette@cancer.dk).

## Supplementary Materials

The following are available online at https://www.mdpi.com/article/10.3390/cancers13184505/s1: Table S1: Grouping of malignant bone tumors in the present study; Table S2: Definition of the 12 main diagnostic groups and 120 disease categories according to the disease codes of the International Classification of Diseases, 7th to 10th revisions (ICD-7 to ICD-10); Table S3: Rate ratios (RRs) and rate differences (RDs) of hospital contact per 1000 person years and 95% confidence intervals (CIs) for first diagnosis of each of the 120 specific disease categories for survivors compared with the matched comparisons.
