# Supplementary material for: Somatic Disease in Survivors of Childhood Malignant Bone Tumors in the Nordic Countries
Source: Cancers (Basel). 2021 Sep 7;13(18):4505. doi: 10.3390/cancers13184505 (PMC8467516; doi:10.3390/cancers13184505)
Supplement: Supplementary file 1 [file cancers-13-04505-s001.zip › cancers-1356317-supplementary.pdf]

Table S1. Grouping of malignant bone tumors in the present study.

	ICCC-1 (ICD-O-1 codes)[a]	ICCC-3 (ICD-O-3 codes)[b]
Osteosarcoma[c]	**VIIIa** (*n* = 242)<br><br>**VIIIb** with the following ICD-O-1 morphology codes:<br>92203 Chondrosarcoma, NOS (*n* = 10)<br>92403 Mesenchymal chondrosarcoma (*n* = 1)<br><br>**VIIIb** with the following C24/histology codes:<br>73.6 (*n* = 21):<br>Chondrosarcoma<br>Chondromyosarcoma<br>Chondrofibrosarcoma<br><br>**VIIId** with the following ICD-O-1 morphology codes:<br>92503 Giant cell tumor of bone, malignant (*n* = 1)<br><br>**VIIId** with the following C24/histology codes:<br>74.6 (*n* = 6):<br>Giant cell tumor of bone, malignant<br>Giant cell sarcoma of bone<br>Osteoclastoma, malignant	**VIIIa** (*n* = 127)<br><br>**VIIIb** with the following ICD-O-3 morphology codes:<br>92203 Chondrosarcoma, NOS (*n* = 24)<br><br>**VIIId/VIIIe** with the following ICD-O-3 morphology codes:<br>92503 Giant cell tumor of bone, malignant (*n* = 2)
Ewing sarcoma	**VIIIc** (*n* = 143)	**VIIIc** (*n* = 37)

[a] ICCC-1 was applied for Denmark (1943–2003) and Sweden (1958–2008) in the ALiCCS-cohort; [b] ICCC-3 was applied for Denmark (2004–2008), Finland (1953–2008), and Iceland (1955–2008) in the ALiCCS-cohort; [c] Including osteosarcoma-like bone sarcomas treated as osteosarcoma.

Table S2. Definition of the 12 main diagnostic groups and 120 disease categories according to the disease codes of the International Classification of Diseases, 7th to 10th revisions (ICD-7 to ICD-10).[a]

Main diagnostic group and disease categories	ICD-7	ICD-8	ICD-9	ICD-10
**Infectious and parasitic diseases**				
Intestinal infectious diseases	040–049	000–009	001–009	A00-A09
Tuberculosis	001–019	010–019	010–018	A15-A19
Sepsis	053	038	038	A40-A41
Erysipelas	052	035	035	A46
Other bacterial diseases	050–051, 054–057, 060–061	030–034, 036–037, 039	030–034, 036–037, 039–041	A30-A39, A42-A45, A47-A49
Enterovirus diseases of CNS	080–083	040–046, 062–066	045–049, 062–064	A80-A81, A83-A89
Herpes zoster	088	053	053	B02
Other viral diseases with exanthema	084–087, 096.0, 096.3	050–052, 054–057	050–052, 054–057	B00-B01, B03-B09
Infectious hepatitis, HIV (only in ICD-9 and 10) and other viral diseases	089, 092–095, 096.1–096.2, 096.4–096.9	070–079	042, 070–079	A70-A71, A82, B15-B34
Syphilis and other venereal diseases	020–039, 070–074	090–104	090–099	A50-A70, A74
Mycoses	121–124	110–117	110–118	B35-B49
Other infectious and parasitic diseases	058–059, 062–064, 090–091,100–120, 125–138	020–027, 060–061, 067–068, 080–089, 120–129, 130–136	020–027, 060–061, 065–066, 080–088, 100–104, 120–136	A20-A28, A75-A79, A90-A99, B50-B83, B85-B99
**Malignant neoplasms (new primary cancer)**				
Cancer of buccal cavity and pharynx				C00-C14, C46.2
Cancer of digestive organs				C15-C26
Cancer of respiratory system and intrathoracic organs				C30-C39, C45.0
Cancer of bones, joints and articular cartilage				C40-C41
Malignant melanoma of skin				C43
Mesothelium and connective tissue				C45.1-C45.9, C46.1, C46.3, C46.7, C46.8, C46.9, C47-C49, B21.0
Cancer of breast				C50
Cancer of female genital organs incl. skin				C51-C58
Cancer of male genital organs incl. skin				C60-C63
Cancer of urinary tract				C64-C68, D30.1-D30.9, D41.4
Cancer of eye, brain and other parts of central nervous system				C69-C72, C75.1-C75.3, D32-D33, D35.2-D35.4, D42-D43, D44.3-D44.5

Cancer of endocrine organs				C73-C74, C75.0, C75.4-C75.9
Malignant lymphomas				C81, C82-C85, C88.3-C88.9
Multiple myeloma				C90, C88.0-C88.2
Leukemia				C91-C96
Ill-defined and unspecified cancer				C76-C80
**Benign neoplasms**				
	210–222, 224–229	210–222, 223.0, 224, 225.5–225.6, 226.0–226.1, 226.4–226.9, 227–228	210–222, 223.0, 224, 225.1, 225.8–225.9, 226, 227.0–227.2227.5–227.9, 228–229	D00-D05, D07-D08, D09.2-D30.0, D31, D34, D35.0-D35.1, D35.5-D35.9, D36
**Endocrine diseases, nutritional deficiencies and other metabolic diseases**				
Diseases of the thyroid gland	250–254	240–242, 244–246	240–242, 244–246	E01-E02, E03.2-E03.9, E04-E07
Diabetes mellitus	260	249, 250	250	E10-E14
Other disorders of glucose regulation and pancreatic internal secretion	270	251	251	E15-E16
Pituitary hypofunction		253.1	253.2–253.3, 253.5	E23.0-E23.3
Ovarian dysfunction	275	256	256	E28
Testicular dysfunction	276	257	257	E29
Disorders of other endocrine organs	271–274, 277	252, 253.0, 253.2–253.9, 254–255, 258	252, 253.0–253.1, 253.4, 253.6–253.9, 254, 255, 258–259, 271	E20-E22, E23.6-E23.9, E24-E27, E30-E35
Nutritional deficiencies	280–286	260–269	260–269	E40-E64
Other metabolic disorders	289	275.4–275.9, 276, 278–279	273, 275, 276, 277.1–277.9	E73, E86-E90
Male sterility	616	606	606	N46
Abnormal menstruation	634	626	626	N91-N92
Female infertility	636	628	628	N97
Other disorders of female reproductive system	635	627	627	N93-N96, N98
**Diseases of blood and blood-forming organs**				
Anemias	290–291, 292.1–292.5, 292.7, 293	280–281, 283–285	280–281, 283–285	D50-D54, D59-D64
Coagulation defects, purpura and other hemorrhagic conditions	295, 296	286–287	286–287	D65-D69
Agranulocytosis	297	288	288	D70-D72
Other diseases of blood and blood-forming organs	299	289	289	D73-D79, D86, D89
**Diseases of nervous system and sense organs**				
Meningitis	340	320	320–322	G00-G03
Other inflammatory diseases of CNS	341–344	321–324	323–326	G04-G09

Multiple sclerosis and other demyelinating diseases of CNS	345	340–341	340–341	G35-G37
Parkinson disease and other movement disorders	350	342	332, 333.1–333.3, 333.5–333.8, 333.90–333.91, 333.93–333.99	G20-G22, G24-G26
Epilepsy	353	345	345	G40-G41
Migraine and other diseases of brain and spinal cord	354–357	346–347, 349	346–349	G13, G43-G44, G46-G47
Senile and presenile dementia		290	290, 331.0	F00-F03, G30-G32
Diseases of nerves and peripheral ganglia	352, 360–369	344, 350–358	350–359	G50-G59, G61-G73, G81-G89, G90.0, G90.2-G90.9, G91-G99
Inflammatory and other diseases of the eye	370–384, 386–389	360–373, 375–379	360–365, 367–379	H00-H22, H30-H36, H40-H59
Cataract	385	374	366	H25-H28
Inflammatory diseases of ear	390–394	380–384	380–384	H60-H75
Meniere’s disease and otosclerosis	395	385–386	386–387	H80-H82
Other diseases of ear and deafness	396–398	387–389	385, 388–389	H83-H95
**Diseases of circulatory system**				
Acute rheumatic fever	400–402	390–392	390–392	I00-I02
Chronic rheumatic heart disease	410–416	393–398	393–398	I05-I09
Hypertensive disease	440–447	400–404	401–405	I10-I15
Ischemic heart disease	420	410–414	410–414, 429.2, 429.7	I20-I25
Pulmonary heart disease	465, 434.0	426, 450	415–417	I26-I28
Pericardial, myocardial and endocardial disease	421, 430–432	420–423	420–423, 424.9, 429.0	I30-I33, I38-I41, I51.4
Valvular disease (non-rheumatic)		424	424.0–424.3	I34-I37
Heart failure	422, 434.1–434.4	425, 427.0–427.1, 428–429	425, 428, 429.1, 429.3, 429.8–429.9	I42-I43, I50, I51.5, I51.7
Conduction disorders	433	427.2–427.9	426–427	I44-I49
Cerebrovascular disease	330–334	430–438	430–438	I60-I69, G45
Diseases of arteries, arterioles and capillaries	450–456, 467.1	440–445, 447–448	440–445, 447–448	I70-I79
Venous and lymphatic disease	460–464, 466, 468	451–457	451–457	I80-I89
Other complications of the circulatory system	467.0, 467.2	446, 458	429.4–429.6, 446, 458–459	I51.0-I51.3, I51.6, I51.8-I51.9, I52, I95-I99, M30
**Diseases of respiratory system**				
Influenza	480–483	470–474	487	J10-J11
Acute upper respiratory infections	470–475	460–465	460–465	J00-J06
Other disorders of upper respiratory tract	240, 510–517	500–508	470–478	J30-J39

Pneumonia	490–493, 525	480–486, 517	480–486	J12-J18
Abscess of lung and pyothorax	518–519, 521	510–511, 513	510–511, 513	J85-J86, J90
Bronchitis and emphysema	500–502, 526, 527.1	466, 490–492, 518	466, 490–492, 494, 496, 518.1–518.2	J20-J22, J40-J44, J47, J98.2-J98.3
Asthma	241	493	493	J45-J46
Lung diseases due to external agents	523–524	515–516	495, 500–508	J60-J70
Interstitial pulmonary diseases and pulmonary edema	522	514, 519.1	514–516, 518.3–518.4	J81-J84
Pneumothorax	520	512	512	J93
Respiratory failure	527.0	519.0	518.0, 518.5–518.8	J96, J98.1
Other diseases of respiratory system	527.2	519.2–519.9	517, 519	J80, J91-J92, J94-J95, J98.0, J98.4-J98.9, J99
**Diseases of digestive organs**				
Diseases of the teeth and supporting structures	530–535	520–525	520–525	K00-K08
Other diseases of the oral cavity and salivary glands	536–538	526–529	526–529	K09-K14
Diseases of esophagus	539	530	530	K20-K23
Diseases of stomach and duodenum	540–545	531–537	531–537	K25-K31
Appendicitis	550–553	540–543	540–543	K35-K38
Hernia of abdominal cavity	560–561	550–553	550–553	K40-K46
Noninfective enteritis and colitis	571, 572	561, 563	555–558	K50-K52
Paralytic ileus and intestinal obstruction	570	560	560	K56
Diseases of anal and rectal regions	574, 575	565, 566	565, 566	K60-K62
Diseases of peritoneum	576, 577	567–568	567, 568	K65-K67
Other diseases of digestive system	573, 578	562, 564, 569	562, 564, 569, 578–579	K55, K57-K59, K63, K90-K93
Diseases of liver	580–583	570–573	570–573	K70-K77
Diseases of gallbladder and biliary ducts	584–586	574–576	574–576	K80-K83, K87
Diseases of pancreas	587	577	577	K85-K86
**Diseases of urinary system and genital organs**				
Glomerular diseases	590–593	580–583	580–583, 599.7	N00-N01, N03-N05, N02.0-N02.8, N06.0-N06.8, N07.0-N07.8, N08
Acute renal failure		593.1	584	N17, N28.0
Chronic kidney disease	594, 600.0	584, 590.0–590.1, 593.0	585–587, 589, 590.0, 590.8, 593.2	N11.8-N11.9, N12, N18-N19, N26-N27, N28.1
Urolithiasis	602, 604	592, 594	592, 594	N20-N22

Obstructive uropathy	601, 608	591, 598, 593.3–593.4, 596.2	591, 593.3–593.5, 596.0, 598, 599.6	N11.0-N11.1, N13.0-N13.5, N13.8-N13.9, N32.0, N35
Infections of the urinary system	605, 607, 600.1–600.2	595, 597, 590.2, 590.9, 599.0	590.1–590.3, 590.9, 595, 597, 599.0	N10, N13.6, N15.1, N16.0, N29.0-N29.1, N30, N33.0, N34, N37.0, N39.0
Other and unspecified disorders of the urinary system	603, 606, 609	593.2, 593.5, 596.0–596.1, 596.3, 596.9, 599.1–599.2, 599.9	588, 593.0–593.1, 593.6–593.9, 596.1–596.9, 599.1–599.5, 599.81–599.89, 599.9	N02.9, N06.9, N07.9, N13.7, N14, N15.0, N15.8-N15.9, N16.1- N16.8, N23, N25, N28.8-N28.9, N29.8, N31, N32.1-N32.9, N33.8, N36, N37.8, N39.1, N39.2-N39.9
Diseases of prostate	610–612	600–602	600–602	N40-N42
Hydrocele and spermatocele	613	603	603	N43
Orchitis and epididymis	614	604	604	N45
Other diseases of male genital organs	615, 617	605, 607	605, 607–608	N44, N47-N51
Chronic cystic disease and other diseases of breast	620–621	610–611	610–611	N60-N64
Inflammatory diseases of female pelvic organs	622–624, 630	612–614, 616.0, 620, 622	614–616	N70-N77
Endometriosis		625.3	617	N80
Noninflammatory disorders of female genital tract	625, 626, 631–633, 637	615, 616.1–616.9, 621, 623–624, 625.0–625.2, 625.9, 629	618–625, 629	N81-N90
**Diseases of skin and subcutaneous tissue**				
Infections of skin and subcutaneous tissue	690–698	680–686	680–686	L00-L08
Other inflammatory conditions of skin and subcutaneous tissue	700–701, 702.0–702.4, 702.6–702.9, 703–708, 711	690–691, 692.0–692.6, 692.8–692.9, 693–698, 708	690–691, 692.00–692.81, 692.83–692.89, 693–698, 702, 708	L10-L57, L59
Radiodermatitis	702.5	692.7	692.82	L58
Disorders of skin appendages (hair, nails, sweat glands)	712–714	703–706	703–706	L60-L75
Other disorders of the skin and subcutaneous tissue	709–710, 715–716	700–702, 707, 709	700–701, 707, 709	L80-L99
**Diseases of bone, joint and soft tissue**				
Arthritis and rheumatism	288, 720–727	274, 710–718	274, 710–719, 725–727, 729.0	M00-M19, M79.0
Osteomyelitis and other diseases of bone and joint	730–738	720–729	720–724, 730–733	M20-M25, M40-M54, M80-M94
Other diseases of musculoskeletal system	740–749	730–738	728, 729.1–729.9, 734–739	M31-M36, M60-M77, M79.1-M79.9, M95-M99

[a]The following chapters in ICD-8 were not included in the analyses: 5 (Psychiatric diseases), 11 (Diseases in pregnancy, during birth and perinatal diseases), 14 (Congenital malformations), 15 (Certain causes of diseases in the perinatal period and death due to this), 16 (Symptoms and ill-defined conditions), 17 (Injuries), and 18 (External cause of accident). Diseases with the following ICD-10 codes were also not included in the analyses: C97 (Cancer arisen independently at several locations), D37-D48 (Non-melanoma skin cancer), C44, C46.0 (Neoplasms of unknown character), E65-E68 (Obesity (ICD-8: 277 and ICD-9: 278)).

Table S3. Rate ratios (RRs) and rate differences (RDs) of hospital contact per 1000 person years and 95% confidence intervals (CIs) for first diagnosis of each of the 120 specific disease categories for survivors compared with the matched comparisons.*.

	Number of hospital contacts by survivors	RR (95% CI)	RD (95% CI)
**Infectious and parasitic diseases**	55	2.28 (1.67 to 3.12)	3.73 (1.91 to 5.56)
Intestinal infectious diseases	13	1.40 (0.76 to 2.58)	0.43 (−0.44 to 1.29)
Sepsis	12	5.39 (2.42 to 12.00)	1.11 (0.33 to 1.90)
Erysipelas	17	9.27 (4.24 to 20.24)	1.75 (0.81 to 2.69)
Other bacterial diseases	8	4.33 (1.71 to 10.98)	0.70 (0.06 to 1.35)
Infectious hepatitis, HIV (only in ICD-9 and 10) and other viral diseases	7	1.25 (0.55 to 2.84)	0.16 (−0.47 to 0.79)
**Malignant neoplasms**	25	3.24 (1.98 to 5.32)	2.00 (0.83 to 3.16)
Cancer of breast	6	4.04 (1.40 to 11.65)	0.51 (−0.04 to 1.07)
Cancer of female genital organs incl. skin	6	2.70 (1.01 to 7.20)	0.43 (−0.14 to 1.00)
**Benign neoplasms**	22	2.05 (1.26 to 3.35)	1.31 (0.20 to 2.41)
**Endocrine diseases, nutritional deficiencies and other metabolic**	25	1.62 (1.04 to 2.54)	1.12 (−0.09 to 2.32)
Diseases of the thyroid gland	5	3.84 (1.22 to 12.10)	0.42 (−0.09 to 0.93)
Diabetes mellitus	5	1.34 (0.50 to 3.57)	0.14 (−0.39 to 0.67)
Abnormal menstruation	5	1.49 (0.55 to 4.02)	0.19 (−0.34 to 0.72)
**Diseases of blood and blood-forming organs**	9	4.03 (1.70 to 9.57)	0.77 (0.09 to 1.45)
Anemias	5	5.37 (1.55 to 18.55)	0.46 (−0.04 to 0.96)
**Diseases of nervous system and sense organs**	25	1.30 (0.84 to 2.01)	0.66 (−0.55 to 1.88)
Diseases of nerves and peripheral ganglia	13	3.19 (1.61 to 6.34)	1.02 (0.19 to 1.85)
Inflammatory diseases of ear	5	1.79 (0.65 to 4.94)	0.25 (−0.27 to 0.78)
**Diseases of circulatory system**	36	2.09 (1.42 to 3.06)	2.19 (0.75 to 3.62)
Pericardial, myocardial and endocardial disease	7	6.27 (2.11 to 18.67)	0.67 (0.07 to 1.26)
Heart failure	9	16.18 (4.38 to 59.75)	0.96 (0.29 to 1.63)
Conduction disorders	6	1.35 (0.55 to 3.29)	0.18 (−0.41 to 0.76)
Cerebrovascular disease	6	3.58 (1.27 to 10.05)	0.49 (−0.07 to 1.05)
Diseases of arteries, arterioles and capillaries	5	5.39 (1.56 to 18.63)	0.46 (−0.04 to 0.97)
Venous and lymphatic disease	6	0.91 (0.38 to 2.17)	−0.06 (−0.66 to 0.53)
**Diseases of respiratory system**	48	1.00 (0.73 to 1.36)	−0.01 (−1.78 to 1.76)
Acute upper respiratory infections	9	0.59 (0.30 to 1.18)	−0.71 (−1.48 to 0.06)

	Number of hospital contacts by survivors	RR (95% CI)	RD (95% CI)
Other disorders of upper respiratory tract	11	0.52 (0.28 to 0.96)	−1.19 (−2.06 to −0.31)
Pneumonia	20	2.29 (1.36 to 3.86)	1.29 (0.25 to 2.33)
**Diseases of digestive organs**	70	1.48 (1.13 to 1.92)	2.76 (0.64 to 4.89)
Diseases of the teeth and supporting structures	15	3.68 (1.91 to 7.10)	1.25 (0.36 to 2.14)
Diseases of esophagus	5	2.99 (1.00 to 8.91)	0.38 (−0.14 to 0.89)
Appendicitis	15	1.04 (0.60 to 1.82)	0.07 (−0.88 to 1.02)
Hernia of abdominal cavity	5	0.65 (0.26 to 1.65)	−0.30 (−0.87 to 0.26)
Noninfective enteritis and colitis	5	0.86 (0.34 to 2.22)	−0.09 (−0.64 to 0.46)
Other diseases of digestive system	9	2.20 (1.01 to 4.78)	0.56 (−0.14 to 1.26)
Diseases of gallbladder and biliary ducts	18	2.11 (1.22 to 3.63)	1.08 (0.09 to 2.07)
**Diseases of urinary system and genital organs**	42	1.62 (1.15 to 2.28)	1.91 (0.31 to 3.51)
Urolithiasis	5	1.92 (0.69 to 5.34)	0.27 (−0.25 to 0.80)
Chronic cystic disease and other diseases of breast	6	1.19 (0.49 to 2.88)	0.11 (−0.48 to 0.69)
Noninflammatory disorders of female genital tract	11	1.40 (0.72 to 2.73)	0.36 (−0.43 to 1.15)
**Diseases of skin and subcutaneous tissue**	36	3.55 (2.34 to 5.39)	3.10 (1.66 to 4.55)
Infections of skin and subcutaneous tissue	17	3.09 (1.70 to 5.60)	1.33 (0.37 to 2.29)
Other inflammatory conditions of skin and subcutaneous tissue	7	2.53 (1.03 to 6.21)	0.49 (−0.13 to 1.10)
Other disorders of the skin and subcutaneous tissue	10	6.79 (2.68 to 17.20)	0.98 (0.26 to 1.70)
**Diseases of bone, joint and soft tissue**	67	1.94 (1.47 to 2.57)	3.98 (1.93 to 6.03)
Arthritis and rheumatism	15	1.41 (0.80 to 2.49)	0.50 (−0.42 to 1.43)
Osteomyelitis and other diseases of bone and joint	41	1.99 (1.39 to 2.84)	2.38 (0.85 to 3.92)
Other diseases of musculoskeletal system	20	2.33 (1.38 to 3.94)	1.33 (0.27 to 2.40)

*Only diseases for which survivors had five or more hospital contacts are shown.
